# Neurophysiological underpinnings of balance control and cognitive-motor interaction in early Parkinson’s disease

**DOI:** 10.1038/s41598-025-06777-1

**Published:** 2025-07-11

**Authors:** Manca Peskar, Paolo Manganotti, Uros Marusic, Klaus Gramann

**Affiliations:** 1 Institute for Kinesiology Research, Science and Research Centre Koper, Garibaldijeva 1, 6000 Koper, Slovenia; 2 Biological Psychology and Neuroergonomics, Department of Psychology and Ergonomics, Faculty V: Mechanical Engineering and Transport Systems, Technische Universität Berlin, Berlin, Germany; 3 Department of Medical, Surgical and Health Sciences, Neurology Unit, Cattinara University Hospital, University of Trieste, Trieste, Italy; 4 Department of Health Sciences, Alma Mater Europaea University, Maribor, Slovenia

**Keywords:** EEG, Semi-tandem stance, COP (center of pressure), Parkinson’s Disease, Cognitive-motor dual-tasking, Cognitive ageing, Cognitive neuroscience, Motor control, Sensorimotor processing

## Abstract

People with Parkinson’s Disease (PD) often compensate for impaired automatic balance control by engaging additional attentional resources for motor tasks. With disease progression, their cognitive system too becomes increasingly affected, further impairing postural stability. The interaction between cognitive and motor systems in the early disease stages, however, remains poorly investigated. The present study aimed to elucidate behavioral and neurophysiological changes in early-stage PD to identify with greater accuracy the specific disease-related discrepancies from healthy functioning on both cognitive and motor systems. Eighteen participants with PD (aged 62.9 ± 6.6 years) and 18 healthy matched controls (aged 62.9 ± 6.4 years) performed (i) a balancing single task in a semi-tandem stance (ST-sts), (ii) a single visual oddball task with conflicting Stroop color-word stimuli (ST-Stroop), and (iii) a dual-task (DT) combining the two single tasks. Centre of pressure displacement using a force plate and 128-channel electroencephalography (EEG) were recorded. Participants with PD exhibited reduced postural sway compared to controls, and postural sway was lower in DT as opposed to ST. Reduced sway in PD might be attributed to postural rigidity and tonic muscle activation, while in the DT, it might reflect resource conflicts. EEG analyses indicated distinct spectral activity patterns: the central midline low-frequency (δ, θ) power increased with cognitive load, centroparietal β desynchronization intensified with motor load, and parietal α desynchronization heightened during DT in both PD and control groups, underscoring specific frequency bands’ governing roles in cognitive-motor processing. Furthermore, PD participants exhibited heightened or prolonged responses in ERP components related to working memory (frontocentral P2) and conflict resolution (P300), possibly reflecting compensatory neural strategies. Overall, these findings suggest that cognitive capacities, particularly selective attention, might be more affected than sensory acuity in early PD, highlighting areas for targeted interventions.

## Introduction

Parkinson’s disease (PD) is the second most common neurodegenerative disorder, estimated to affect 3% of people aged 65 and up to 10% of people over the age of 80^1,2^. The disease has been linked to the loss of the dopamine neurons in the Substantia nigra pars compacta and is manifesting most severely in motor symptoms, such as tremors at rest, rigidity, bradykinesia, and postural instability^3–5^.

Postural stability, defined as the ability to keep the body’s center of mass inside the base of support, deteriorates with aging, which is even more pronounced in people with PD^6^. Failing to ensure postural stability might result in falling, and a prospective study showed that PD patients are three times more likely to fall compared to age-matched healthy controls^7^. When measuring postural sway during balancing in an upright stance, however, studies are inconclusive; some report increased postural sway in PD patients compared to healthy controls^8–14^, while others show no difference^15–17^ or less postural sway in PD compared to healthy controls^18,19^, offering no clear indication of how much postural sway is optimal for flexible motor adjustments/control. These findings demonstrate that behavioral outcomes fail to provide a reliable approach for the differentiation of healthy and impaired balance. The investigations into brain dynamics related to cortical control of balance might offer additional insights.

Previous studies in neurotypical individuals using electroencephalography (EEG) to investigate the brain dynamics underlying balance control showed increased theta (4 - 7 Hz) spectral power in the supplementary motor area when introducing a more challenging tandem stance (heel-to-toe) compared to the regular stance (feet parallel;^20^). A robust increase in theta activation over the sensorimotor and occipital cortices was also observed upon the addition of a secondary visual oddball task to a regular stance balancing, forming a cognitive-motor dual-tasking condition in older participants^21^. The increase in postural demands associated with tandem compared to the regular stance, in addition, promoted widespread mu (8 - 12 Hz) and beta (13 - 30 Hz) suppression in the old compared to the young group^20^. Similar spectral power changes in theta, alpha (8 - 12 Hz), and beta bands were observed while walking on a balance beam as compared to the flat treadmill belt in the anterior cingulate, posterior parietal, dorsolateral-prefrontal, and sensorimotor cortices^22^.

Suppression or desynchronization of sensorimotor beta activity is strongly implicated in motor planning and flexibility in motor execution in a wide range of motor tasks spanning from simple finger-tapping to full-body overground walking^23–27^. One of the characteristic features of PD, in contrast, is increased synchronization within the beta band in neurons of the subthalamic nucleus (part of the motor circuit), which is associated with the severity of bradykinesia^28,29^. It remains poorly understood how the seemingly contradictory roles of beta desynchronization and synchronization in motor control converge in patients with PD, particularly during static balancing in both single-task and cognitive-motor dual-task scenarios.

People with PD typically compensate for the lack of automaticity in balance control by employing additional attentional strategies^30^. However, this compensation can be impeded as the cognitive system itself becomes affected by the disease. Cognitive impairment has been observed in 1 out of 3 patients already in their early disease stage (early PD), leading to attentional, executive function, language, and visuospatial deficits^31,32^. Since PD is primarily characterized by motor symptoms, the non-motor plethora of symptomatology, including cognitive impairments, can often be overlooked, especially in the early disease stage. However, the neurophysiological bases of cognitive function, such as visual processing and attention, can be elegantly investigated with electroencephalography (EEG) using the magnitude and timing of event-related potentials (ERP) and their components (e.g., N1, P2, N2, P300). For instance, in Alzheimer’s disease, it is well documented that the peak P300 typically occurs later and with a smaller amplitude compared to healthy individuals^33^. While there is some indication of similar trends being present in PD, the evidence is not as convincing or was acquired in people in later disease stages or suffering from PD-related dementia^33,34^.

Overall, there is a knowledge gap about how people with PD control static balance by engaging their cognitive resources if the standing task requires more control than the simple regular stance (e.g., the semi-tandem) and if a secondary cognitive task is to be performed in parallel. The cognitive-motor dual-tasking paradigms offer an increased ecological validity to the observed effects and are inherently important for understanding the complex disease manifestations, such as PD, which affects not only the motor but also the cognitive system.

This study investigated behavioral (postural sway) and EEG (spectral power, ERP) characteristics of early PD participants and age-matched healthy controls performing a static semi-tandem postural task with and without the addition of a secondary visual oddball task. Our goals were to elucidate in both healthy and people in the early stages of PD (i) the postural sway using centre of pressure (COP) displacement during semi-tandem stance, (ii) the spectral power features characterizing motor, cognitive, and cognitive-motor investment, and (iii) the neurophysiological markers of sensory visual and cognitive function reflecting cognitive single-tasking and cognitive-motor dual-tasking as detected by ERP components. We hypothesized to observe less desynchronization in the beta band in participants with PD compared to healthy controls during tasks that include motor components, and increased theta synchronization upon introduction of the dual-task as compared to the single-motor task, as well as prolonged response and/or lower amplitude of P300 in PD compared to healthy controls.

## Methods

### Participants

Of the initially recruited 22 participants with early-stage PD and 27 healthy participants, 18 participants with PD (mean age *M* = 63.4 ± 6.6 years; 8 women) and 18 age- and sex-matched healthy participants (mean age *M* = 63.0 ± 6.7 years; 7 women) participated in the study (drop out due to withdrawn consent, technical problems during data collection, and the inability to perform the task). Participants’ characteristics are displayed in Table 1. Inclusion criteria for the participants were (1) Montreal Cognitive Assessment (MoCA) score ≥ 24 points; (2) Short Physical Performance Battery (SPPB) score ≥ 8 points; (3) age range between 50 and 75 years, (4) no color blindness, (5) living independently in the community. Additionally, inclusion criteria for the healthy control group encompassed (6) no diagnosis of cognitive or movement disorder, while for the participants with PD, the following were considered: (6) diagnosed according to the last International Movement Disorder Society Unified Parkinson’s Disease Rating Scale (MDS-UPDRS) criteria (2015), (7) duration of the disease less than 5 years, (8) score of 1 or 2 on the Hoehn and Yahr scale. All participants had normal or corrected-to-normal vision and were right-handed. Healthy controls were recruited by word of mouth and through the Research Centre’s database, while the participants with PD were recruited via the Clinical Unit of Neurology, University of Trieste.

**Table 1:** Participants’ characteristics separated per group.

	Healthy Controls	Participants with PD	*p*
Age (*M* ± *SD*) [years]	62.9 ± 6.36	63.4 ± 6.59	0.818
Height (*M* ± *SD*) [cm]	174 ± 7.1	171 ± 8.2	0.197
Weight (*M* ± *SD*) [kg]	79.8 ± 12.8	72.5 ± 16.1	0.143
Education (*M* ± *SD*) [years]	15.5 ± 4.19	12.3 ± 3.95	0.023
SPPB Score (*M* ± *SD*) [points]	11.9 ± 0.35	9.8 ± 1.48	< 0.001
MoCA Score (*M* ± *SD*) [points]	27.6 ± 1.72	27.7 ± 1.85	0.853
MDS-UPDRS (*M* ± *SD*) [points]	na	29.2 ± 14.4	
Number of women	7	8	
Number of retired participants	9	9	
Partnership status			
Married	10	16	
Extramarital union	6	1	
Divorced	1	0	
Single	1	1	
Number of smokers	1	1	
Number of fallers in the last year	6	3	
Number of drug-treated comorbidities			
Hypertension	4	8	
Diabetes	0	1	
Cardiac condition	2	2	
Metabolic condition	0	5	
Rheumatism	2	5	
Depression	0	3	

SPPB : Short Physical Performance Battery, MoCA : Montreal Cognitive Assessment, MDS-UPDRS: Movement Disorder Society: United Parkinson’s Disease Rating, na : non-applicable.

### Ethics & consent

The study was conducted in accordance with the ethical standards outlined in the 1964 Declaration of Helsinki and the guidelines of Good Clinical Practice. The Institutional Review Board (IRB) of Trieste University Hospital: ASUGI, Trieste, Italy, approved the study protocol (ASUGI protocol number: 106/2021). The study was registered on ClinicalTrials.gov under the code NCT05477654. Written informed consent was obtained for each study participant. All participant information and data were securely stored and identified solely by coded ID numbers to ensure participants’ confidentiality.

### Procedure and study design

This report forms part of a larger study (see study protocol:^35^). Upon volunteering, potential participants initially underwent a telephone screening to confirm age and health eligibility. For patient participants, their health status was clinically evaluated prior to participant recruitment. Both participant groups were subsequently invited to an in-person screening session (M1), where they provided written informed consent, familiarized themselves with the study procedures, and performed baseline assessments. These assessments included a demographic and health questionnaire, the MoCA, the SPPB, and, for participants with Parkinson’s disease (PD), the Movement Disorder Society-Unified Parkinson’s Disease Rating Scale (MDS-UPDRS). Further descriptions of these measures can be found under the Secondary Measures section. Participants who met the inclusion criteria were then invited to the measurement session (M2).

**Fig. 1:**
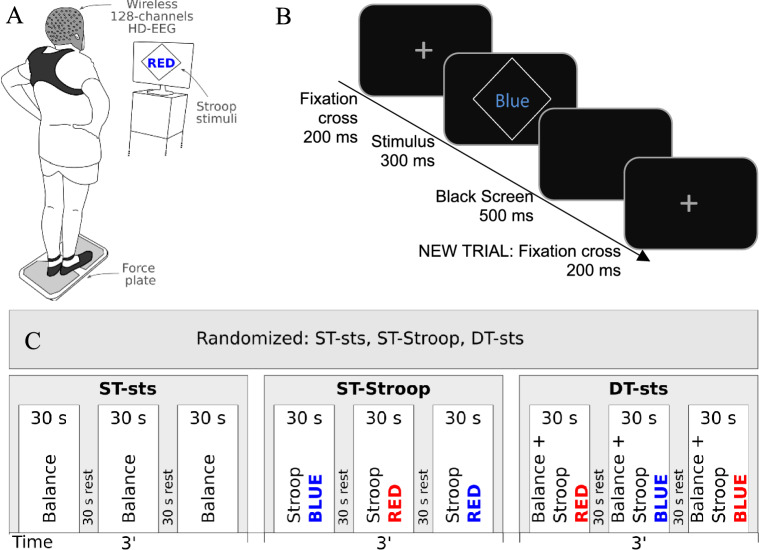
Setup (**A**), Trial Sequence (**B**), and Task condition order and duration (**C**) in the Balancing Experiment. Abbreviations: ST: single task, DT: dual task, sts: semi-tandem stance, Stroop: Stroop stimuli counting task.

On M2, we recorded EEG during (i) the resting state while seated with eyes open and closed, at both the beginning and the end of the session (> 3 min each), (ii) quiet standing with eyes open (> 2 min), (iii) and the balancing experiment. In this experiment, participants maintained the semi-tandem stance position in the single task (ST-sts), performed a cognitive single task (ST-Stroop) in the seated position, and performed the dual-task (DT-sts) condition (maintaining semi-tandem stance while performing the cognitive task). Fig. 1 depicts the setup and the task conditions’ order.

This study employed a mixed between-within factorial design with 2 [group: healthy vs PD] × 3 [condition: ST-sts vs. ST-Stroop vs. DT-sts] levels. For some outcome measures, a reduced 2 × 2 mixed design was applied, as specified below.

### Secondary measures

The Montreal Cognitive Assessment (MoCA;^36,37^) is a screening tool for early detection of mild cognitive impairment. It assesses visuospatial and executive function, memory, language, attention, abstraction, and orientation. The maximum score is 30 points.

The Short Physical Performance Battery (SPPB;^38,39^) is used to assess lower extremity function, particularly balance, gait, strength, and endurance. The maximum score is 12 points while the generally accepted cutoff score to detect frailty is 8.

The Movement Disorder Society: Unified Parkinson’s Disease Rating Scale (MDS-UPDRS;^40^) is a tool to evaluate symptoms of Parkinson’s Disease, including motor and non-motor experiences of daily living and motor complications.

### Task description, stimuli & behavioural measures

The Task Description relates to the measurement day M2. In the single-task balancing condition (ST-sts), participants were asked to stand straight and still on a force plate (BTrackS™ Balance Plate) in a semi-tandem stance with their hands on their hips. They were free to choose which foot to put in front/back, but had to stick with the decision throughout the experiment. Balancing runs were performed three times for 30 seconds each, while the participants fixated on a cross displayed on the presentation screen. Between the runs there were 30-second breaks. The force plate recorded the metrics quantifying the center of pressure (COP) magnitude and derivatives (path length: PL [cm]; Ellipse area fitting the smallest 95% of the COP trace: ELL [cm^2^]; Average COP velocity in a run: VEL [cm/s];^41,42^).

In the single-task Stroop condition (ST-Stroop), participants sat in a chair and were asked to silently count the number of occurrences of specific Stroop stimuli. We used red and blue colors, leading to 2 congruent and 2 incongruent word-color combinations. Across the three 30-second repetitions, participants had to count the occurrences of the word »Blue« written in blue ink, followed by the word »Red« written in red ink, and lastly, the word »Red« written in blue ink, respectively (target trials), among the other three non-target stimuli combinations (regular trials). A trial began with a fixation cross displayed for 200 ms, followed by a stimulus presentation for 300 ms, and a blank screen for 500 ms. The inter-stimulus interval was fixed at 1s, leading to a presentation of 30 successive stimuli within a 30-second run. The number of target stimuli within the first and the third run was 8 (26.6%), whereas in the second it was 7 (23.3%), leading to a total number of 23 target stimuli for the ST-Stroop task. The ratio between target and non-target stimuli corresponded to the classical oddball paradigm (20–30% of target stimuli). The verbally reported number of stimulus occurrences at the end of each run provided an indirect behavioral measure of success.

In the dual-task condition (DT-sts), the above-described tasks - ST-sts and ST-Stroop, were performed in parallel across the three 30-second runs. The specifications of the Stroop stimuli that had to be counted across the runs were as follows: occurrences of the word »Red« written in red ink, followed by the word »Blue« written in blue ink, and lastly the word »Blue« written in red ink. The number of target stimuli within the first and the third run was 7 (23.3%), whereas in the second it was 8 (26.6%), leading to a total of 22 target stimuli in the DT-sts task. The other specifics and outcome measures were kept the same as in the ST conditions.

In all conditions, the presentation screen was positioned 110 cm from the participant’s eyes and at their eye-level height. Between the three conditions, there was approximately 1-min break. The three conditions were randomized across participants.

### EEG acquisition and analysis

EEG was recorded using a wireless CGX Mobile-128 channel system (Cognionics Inc., San Diego, CA, USA) using 128 active wet Ag/AgCl scalp electrodes mounted in an elastic EEG cap and positioned according to the 10–5 montage system^43^. Reference and ground electrodes were placed on the right and left mastoids, respectively. Before recording, the electrode impedance was brought below 20 kΩ for each channel. Data were recorded at a sampling rate of 500 Hz using a DC-coupled amplifier with 131 Hz high cut-off, and digitized at 24 bits of resolution.

Data were processed using custom semi-automated MATLAB scripts (The MathWorks, Inc.) that included EEGLAB^44^, and ERPLAB^45^ toolboxes (similar to^46^). All continuous resting-state and task-related recordings of single participants were concatenated into a single data file, downsampled to 256 Hz, high-pass filtered at 1 Hz, had 50 Hz line noise removed using the ZapLine method^47^, underwent automatic bad channel detection using *clean_artifacts* algorithm (*FlatLineCriterion* = 10 s, *ChannelCriterion* = 0.80, *ChannelCriterionMaxBadTime* = 0.5), and had bad channels interpolated using a spherical spline interpolation. In a subsequent visual inspection, additional bad channels were removed manually and interpolated. On average, 13.0 ± 8.2% and 10.5 ± 6.5% of channels were interpolated for the healthy and participants with PD, respectively. The data were re-referenced to the average reference. Then, all single-subject data were inspected in the time domain when segments of data displaying muscle artifacts or other major disruptions were manually rejected; on average, a concatenated single-subject recording was 1271 ± 224 seconds long of which 15.9 ± 10.1% were removed. Finally, the Adaptive Mixture Independent Component Analysis (AMICA;^48^) was applied (*max_iter* = 2000, *max_threads* = 4; data rank reduced by the number of interpolated channels plus one accounting for average re-referencing). For the resultant independent components (ICs), equivalent dipole models were computed using the DIPFIT plugin with default settings^49^. All ICs were labelled using the ICLabel plugin^50^. Independent components labelled »eye«, »muscle«, » line-noise «, »channel noise«, or »other« exceeding a threshold of 30% were rejected. This resulted in the removal of *M* = 13.5 ± 6.1 ICs per subject, which did not differ between the groups (*p* = *0.573*).

Following the preprocessing, data time-locked to the Stroop stimuli onset were epoched to [-200 800] ms intervals and epochs with channels exceeding 150 μV were rejected (on average, 2 ± 7.2 and 3.6 ± 7.8 trials were rejected in ST-Stroop and DT-sts, respectively). Single-subject EPRs were computed by averaging responses to the target and non-target Stroop stimuli categories separately for each condition (ST-Stroop, DT-sts).

The time windows for extraction of the peak amplitudes and latencies were determined on the grand average waveform as symmetrical intervals between the approximate x-axis zero crossing. The following time windows were used: for early visual components extracted from the Oz electrode - N1 [90 190], P2 [180 280]; for frontocentral components extracted from the FPz electrode -  P2 [100 200], N2 [220 320]. The P300 mean amplitudes were extracted from the Pz electrode across four consecutive 100-ms-long time windows spanning across 300–700 ms post-stimulus.

For the spectral analysis, we estimated the power spectral density of 2-second windows of segmented data epochs with 1-second overlap at all channels. The canonical frequency bands were defined as: δ (1.5–3.5 Hz), θ (4–7.5 Hz), α (8–12 Hz), β (12.5–30 Hz), γ-low (30.5–50 Hz), γ-high (50.5–70 Hz). The average power within the respective frequency bands was calculated at a single-subject level at midline electrodes Fz, Cz, Pz, and Oz.

### Statistical analysis

Statistical analyses were performed with SPSS software version 28.0 (IBM, Chicago, IL) using a repeated measures ANOVA (rmANOVA) design. Postural sway measures (PL, ELL, VEL) were subjected to 2 (group: healthy vs. PD) × 2 (task: ST-sts vs DT-sts) mixed between-within rmANOVA design; ERP measures (peak amplitude and latency *or* mean amplitude) were subjected to 2 (group: healthy vs. PD) × 2 (task: ST-Stroop vs DT-sts) × 2 (Stimulus: target vs regular) design; spectral analysis measures were subjected to 2 (group: healthy vs. PD) × 3 (task: ST-sts vs ST-Stroop vs DT-sts) design. To address violations of the sphericity assumption (covariance), the Greenhouse–Geisser correction was applied, denoted as (GG). In the event of a significant interaction, simple effects were examined. The alpha level was maintained at 0.05. However, for post hoc pairwise comparisons, a Bonferroni correction was used to minimize the risk of false positives. Effect sizes are presented as adjusted partial eta squared (*adj* ${\upeta }_{\text{p}}^{2}$; ^51,52^). The relationships between behavioral force plate data and neurodynamic ERP and spectral power markers were assessed using Pearson correlation coefficients.

## Results

### Behavioral measures: stroop counts, COP displacement

The behavioral performance on the Stroop stimuli counts is presented in Table 2.

**Table 2:** Description of Stroop counting error occurrences separated by group and task.

	Single-task Stroop counts		Dual-task Stroop counts	
	#	Degree of deviation	#	Degree of deviation
Healthy	1	(− 1)	3	(− 1, − 2, − 2)
PD	6	(+ 2, + 2, − 1, − 1, − 2, − 3)	3	(− 1, − 2, − 3)

#: number of people reporting incorrect counts; degree of deviation: characterizes mistakes in counting for each participant (number shows the distance from the correct sum;—and + show direction of error: fewer and more counts than correct, respectively).

**Fig. 2:**
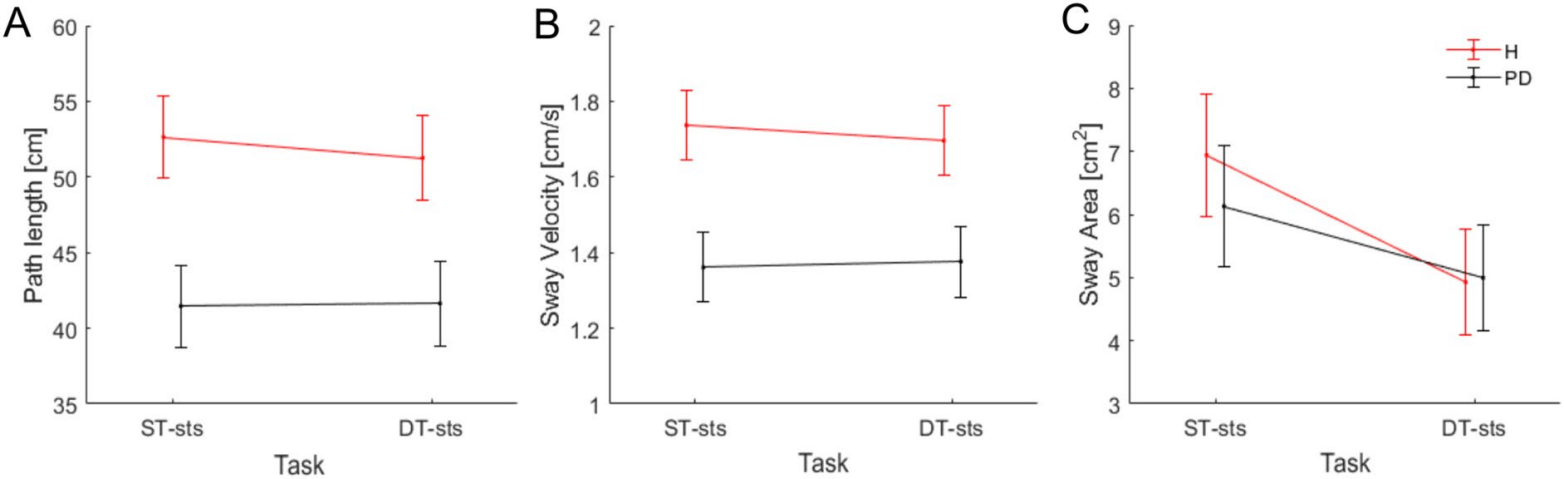
Centre of pressure (COP) displacement measures separated by group and task for (A) Path Length, (B) Sway Velocity, and (C) Sway area. The vertical bars denote Standard Errors (SE). Abbreviation: H: healthy , PD: Parkinson’s Disease.

The rmANOVA for the COP displacement at a between-subject level showed a main effect of group on path length (*p* = *0.009*) revealing less sway in PD (*M* = 41.5, *SE* = 2.7) compared to healthy participants (*M* = 51.9, *SE* = 2.7), and a main effect of group on velocity (*p* = *0.008*) pointing to slower sway in PD (*M* = 1.37, *SE* = 0.09) compared to healthy participants (*M* = 1.72, *SE* = 0.09), while at a within-subject level, we observed a main effect of task on sway area (*p* < *0.001*) indicating less sway in DT-sts (*M* = 4.96, *SE* = 0.59) compared to ST-sts (*M* = 6.54, *SE* = 0.59). The results on COP displacement separated per group and task are depicted in Fig. 2.

### Neurophysiological measures: spectral power

**Fig. 3:**
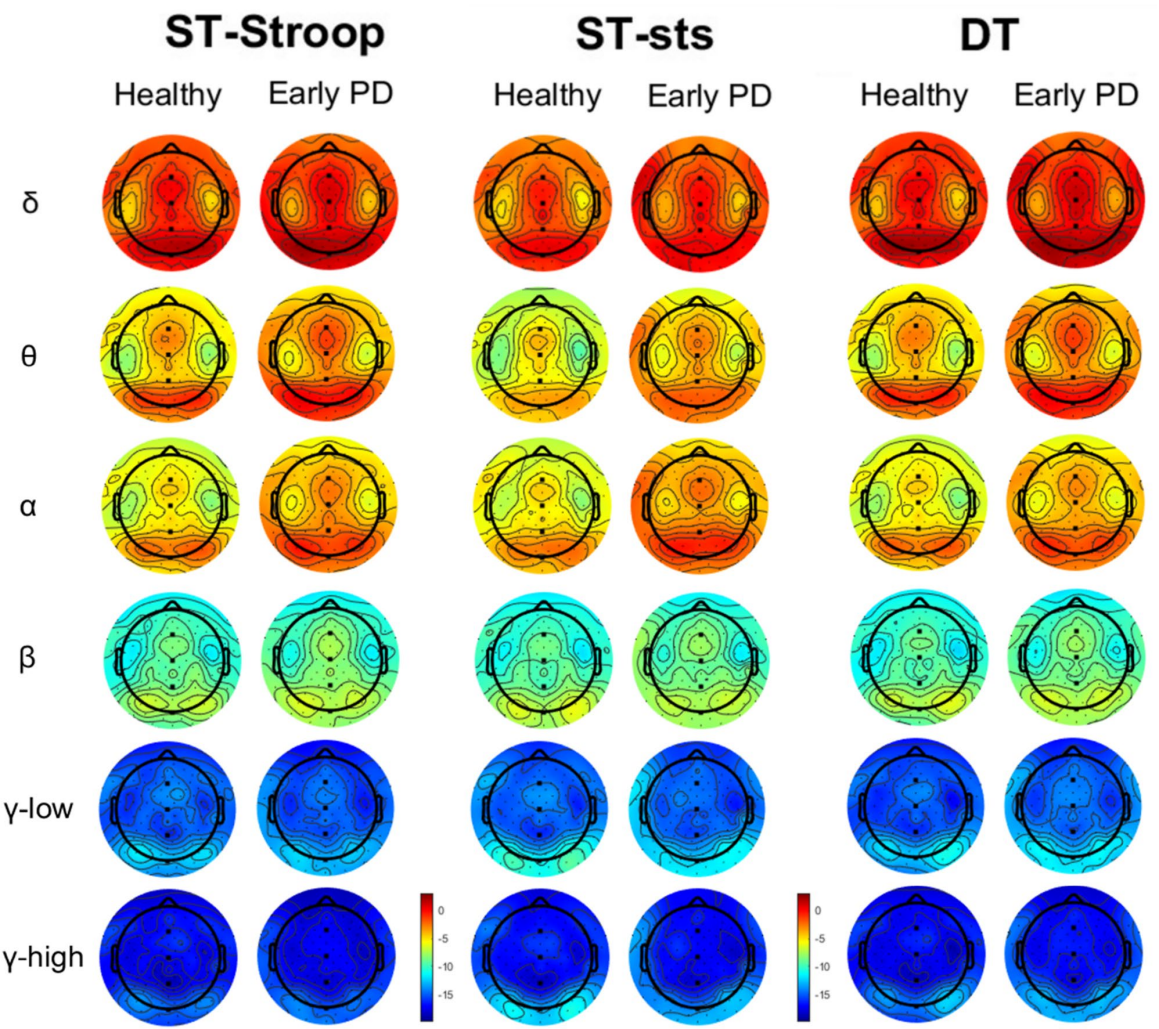
Power Spectral Density (PSD) across groups, task conditions, and frequency bands. The values are color-coded and expressed in dB, range [-19 3]. Abbreviation: δ: delta, θ: theta, α: alpha, β: beta, γ-low: low gamma, γ-high: high gamma, ST—single-task, DT—dual-task, sts—semi-tandem stance, Stroop—Stroop stimuli counting task. The Figure was created using MATLAB (R 2022a, version: 9.12.0), The MathWork, Inc.

The results of the 2 × 3 rmANOVA for the four midline electrodes (Fz, Cz, Pz, Oz) and six frequency bands (δ, θ, α, β, γ-low, γ-high) are comprehensively presented in Table S1, summarized in Table 3 (for *p* < 0.1) and depicted in Fig. 3.

**Table 3:** Mixed between-within ANOVA results for the spectral power of six frequency bands.

SITE	FREQ	FACTOR	*df*_*1,*_* df*_*2*_	F	*p*		*adj* ${\upeta }_{\text{p}}^{2}$
Fz	δ	Task	2, 68	15.3	< 0.001		0.291
	θ	Task	1.4, 48	38.3	< 0.001	_(GG)_	0.515
Cz	δ	Task	2, 68	4.81	0.011		0.098
	θ	Task	1.5, 50.4	17.3	< 0.001	_(GG)_	0.317
	β	Task	2, 68	3.15	0.049		0.058
Pz	δ	Task	2, 68	12.1	< 0.001		0.241
	θ	Task	1.4, 49.1	16.7	< 0.001	_(GG)_	0.311
	α	Task	1.5, 50	4.5	0.025	_(GG)_	0.092
	β	Task	2,68	10.4	< 0.001		0.213
Oz	δ	Task	1.7, 56.9	10.54	< 0.001	_(GG)_	0.214
	θ	Task	1.3, 43.2	25.84	< 0.001	_(GG)_	0.420

FREQ.: frequency band, *adj* ${\upeta }_{\text{p}}^{2}$: adjusted partial eta squared, δ: delta, θ: theta, α: alpha, β: beta, γ-high: high gamma, G*T: group*task interaction, GG: Greenhouse Geiser corrected. only results with *p* < 0.05 are presented; see Table S1 for a comprehensive display.

In the δ band, we observed an effect of task at all four midline electrodes (all *p* ≤ *0.011*). Bonferroni-corrected pairwise comparisons revealed lower PSD in ST-sts (*M* = -0.82, *SE* = 0.56) compared to both ST-Stroop (*M* = 0.17, *SE* = 0.58, *p* < 0.001) and DT (*M* = 0.30, *SE* = 0.58; *p* < 0.001) at Fz, lower PSD in ST-sts (*M* = -0.66, *SE* = 0.50) compared to both ST-Stroop (*M* = 0.11, *SE* = 0.48; *p* = 0.007) and DT (*M* = 0.28, *SE* = 0.48;* p* < 0.001) at Pz, lower PSD in ST-sts (*M* = 0.33, *SE* = 0.57) compared to both ST-Stroop (*M* = 1.55, *SE* = 0.62; *p* < 0.001) and DT (*M* = 1.80, *SE* = 0.66; *p* = 0.003) at Oz, and lower PSD in ST-sts (*M* = 0.12, *SE* = 0.63) compared to DT (*M* = 1.01, *SE* = 0.61; *p* = 0.020) at Cz.

In the θ band, an effect of task was observed at Fz, Cz, Pz, and Oz (all *p* < 0.001) and Bonferroni-corrected pairwise comparisons showed that at all sites, the PSD in ST-sts was lower compared to both ST-Stroop and DT (Fz: ST-sts (*M* = -3.80, *SE* = 0.66), ST-Stroop (*M* = -2.35, *SE* = 0.62), DT (*M* = -2.20, *SE* = 0.62); Cz: ST-sts (*M* = -3.61, *SE* = 0.70), ST-Stroop (*M* = -2.54, *SE* = 0.66), DT (*M* = -2.37, *SE* = 0.65); Pz: ST-sts (*M* = -4.25, *SE* = 0.63), ST-Stroop (*M* = -3.36, *SE* = 0.57), DT (*M* = -3.36, *SE* = 0.57); Oz: ST-sts (*M* = -2.97, *SE* = 0.56), ST-Stroop (*M* = -0.85, *SE* = 0.63), DT (*M* = -0.67, *SE* = 0.65); all *p* < 0.001).

In the α band, we observed an effect of task at Pz (*p* = *0.025*) with Bonferroni-corrected pairwise comparisons revealing lower PSD in DT (*M* = -3.93, *SE* = 0.79) compared to both ST-Stroop (*M* = -3.51, *SE* = 0.81; *p* = 0.027) and ST-sts (*M* = -3.20, *SE* = 0.89; *p* = 0.042). In the α band at the Pz electrode, we also observed a trend for the group*task interaction at (*p* = 0.096), which has not been explored further.

For the β band, we observed an effect of task at Pz electrode (*p* < 0.001) with Bonferroni corrected pairwise comparisons showing higher PSD in ST-Stroop (*M* = -8.67, *SE* = 0.67) compared to both ST-sts (*M* = -9.19, *SE* = 0.66; *p* = 0.003) and DT (*M* = -9.13, *SE* = 0.64; *p* = 0.001). A similar effect, however failing to survive Bonferroni correction, was observed at the Cz electrode (*p* = 0.049) suggesting higher PSD in ST-Stroop (*M* = -8.32, *SE* = 0.72) compared to both ST-sts (*M* = -8.85, *SE* = 0.68; *p* = 0.092) and DT (*M* = -8.79, *SE* = 0.65; *p* = 0.056). In addition, a trend for an effect of task in the β band was observed at Fz electrode (*p* = 0.082), suggesting higher power in ST-Stroop (*M* = -8.36, *SE* = 0.70) compared to both ST-sts (*M* = -8.63, *SE* = 0.72; *p* = 0.135) and DT (*M* = -8.66, *SE* = 0.66; *p* = 0.126), but not for the comparison between ST-sts and DT (*p* = 1.000).

In low and high gamma bands, no significant effect was observed apart from a trend for an effect of task (*p* = 0.080) in the high gamma band at the Oz electrode suggesting higher power in the ST-sts (*M* = -13.6, *SE* = 0.70) compared to ST-Stroop (*M* = -15.0, *SE* = 0.78; *p* = 0.130). Figure 3 depicts topographical representations separated by condition, group, and frequency band.

### Neurophysiological measures: ERP results

**Fig. 4:**
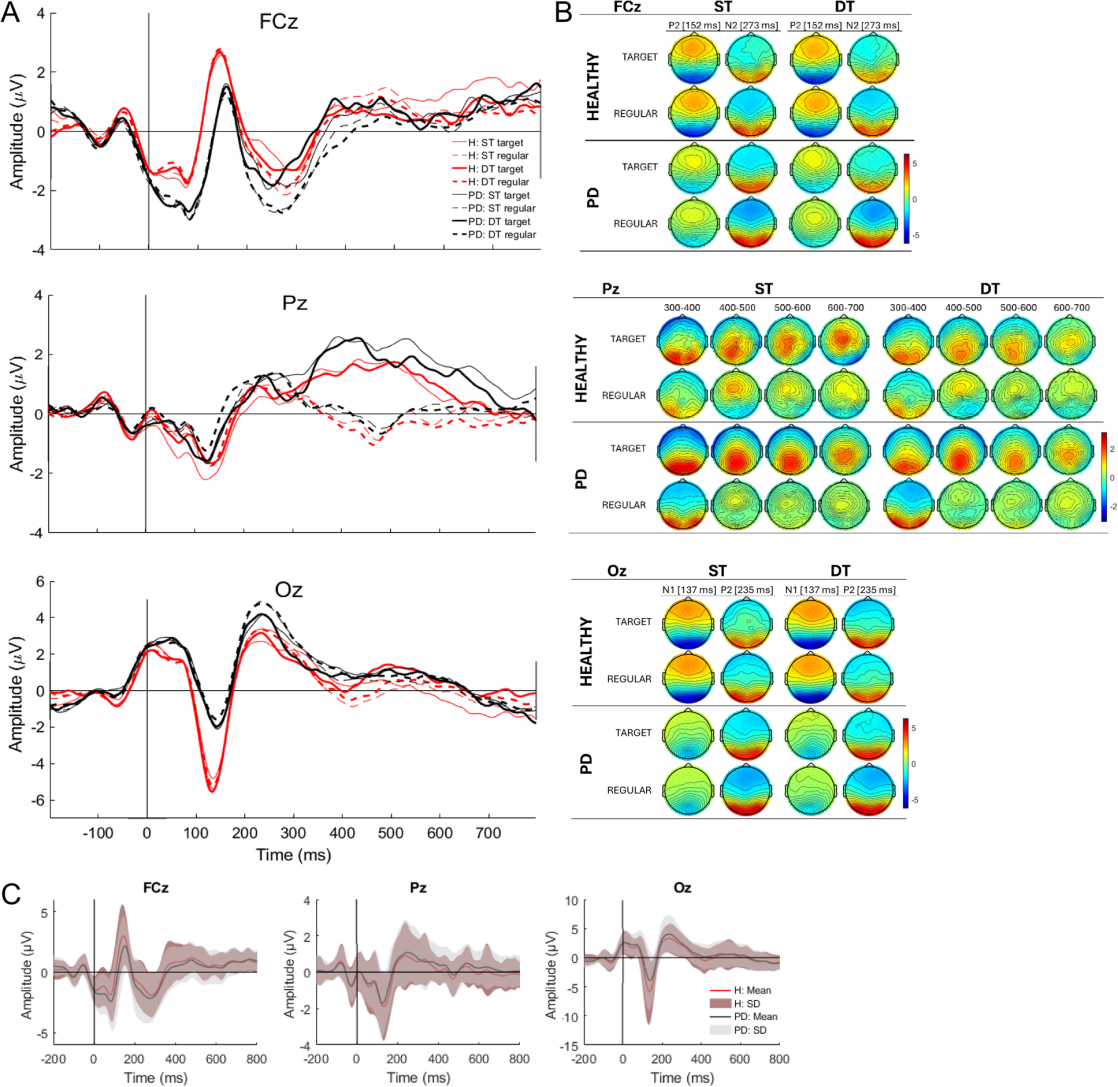
Stroop-count related ERPs. (**A**) ERPs across FPz (top), Pz (middle), and Oz (bottom) separated by group (red: healthy controls, black: participants with PD), task complexity (thin line: ST, bold line—DT), and stimulus type (solid line: target, dashed line—regular), and (**B**) their respective topographies at the grand average ERP peak latencies are displayed for FCz (top) and Oz (bottom) and the mean amplitude values across 300–700 ms grouped in four 100 ms time windows for the Pz (middle). (**C**) Group averaged ERPs (lines) with standard deviations (shaded area) are presented across the FCz (left), Pz (middle), and Oz (right) electrodes. The Figure was created using MATLAB (R 2022a, version: 9.12.0), The MathWork, Inc.

The results of the 2 × 2 × 2 mixed within-between rmANOVA for the Stroop-count-related ERP amplitude and/or latency values are comprehensively presented in Table S2, summarized in Table 4 (for *p* < 0.1), and depicted in Fig. 4.

**Table 4:** Mixed between-within rmANOVA results for the amplitude and latency values of the ERPs.

SITE	COMPONENT	FACTOR	F	*p*	*adj* ${{\varvec{\eta}}}_{{\varvec{p}}}^{2}$
Oz	N1 amplitude	Task	4.23	0.047	0.085
		T*S	8.37	0.007	0.173
FCz	P2 amplitude	G*S	5.04	0.031	0.103
	P2 latency	Group	11.1	0.002	0.224
		Stimulus	6.41	0.016	0.134
	N2 amplitude	Stimulus	5.44	0.026	0.113
	N2 latency	T*S	4.76	0.036	0.097
Pz	MA 300–400	Stimulus	30.4	< 0.001	0.456
	MA 400–500	Stimulus	87.6	< 0.001	0.712
	MA 500–600	Stimulus	81.7	< 0.001	0.697
	MA 600–700	Group	4.86	0.034	0.099
		Stimulus	27.2	< 0.001	0.429

In all cases, *df*_*1*_ = 1, and *df*_*2*_ = 34. *adj* ${\upeta }_{\text{p}}^{2}$: adjusted partial eta squared, G*T: group*task interaction, G*S: group*stimulus interaction, T*S: task*stimulus interaction. only results with *p* < 0.05 are presented; see Table S2 for a comprehensive display.

For the occipital N1 amplitude, we observed a main effect of task (*p* = 0.047) indicating diminished negativity for the ST (*M* = -4.95, *SE* = 0.77) as compared to the DT (*M* = -5.25, *SE* = 0.77), but the task*stimulus interaction (*p* = 0.007) and Bonferroni-corrected pairwise comparisons revealed that this effect was only observed for the target stimuli (in ST: *M* = -4.79, *SE* = 0.77; in DT: *M* = -5.50, *SE* = 0.78; *p* = 0.008), and not for the regular stimuli (in ST: *M* = -5.11, *SE* = 0.79; in DT: *M* = -5.00, *SE* = 0.77; *p* = 0.420). In addition, the interaction showed greater negativity for DT target compared to DT regular stimulus (*p* = 0.012), but this was not observed for the target and regular stimuli of the ST (*p* = 0.167).

The frontocentral P2 amplitude displayed group*stimulus interactions (*p* = 0.031), and the Bonferroni-corrected pairwise comparisons showed greater amplitude for the target (*M* = 2.60, *SE* = 0.53) compared to the regular (*M* = 2.13, *SE* = 0.52), stimulus in the PD group (*p* = 0.011), but not in the healthy group (target: *M* = 3.19, *SE* = 0.53; regular: *M* = 3.27, *SE* = 0.52; *p* = 0.625). Similarly, no effects were detected for the target stimulus across groups (*p* = 0.441) nor the regular stimulus across groups (*p* = 0.129).

The frontocentral P2 latency showed an effect of stimulus (*p* = 0.016), indicating longer latencies for the target (*M* = 150.6, *SE* = 2.23) than for the regular (*M* = 148.2, *SE* = 2.46) stimuli. The frontocentral P2 latency also showed sensitivity for differentiating groups (*p* = 0.002) with longer latencies observed in the PD (*M* = 157.0, *SE* = 3.25) compared to the healthy (*M* = 141.7, *SE* = 3.25) group.

The frontocentral N2 amplitude showed an effect of stimulus (*p* = 0.026), indicating greater negativity for the regular (*M* = -2.90, *SE* = 0.35) compared to the target (*M* = -2.45, *SE* = 0.37) stimulus. The frontocentral N2 latency showed a task*stimulus interaction (*p* = 0.036), however, the effects did not survive the Bonferroni pairwise correction (target in ST: *M* = 270.5, *SE* = 6.25; regular in ST: *M* = 263.8, *SE* = 4.21; target in DT: *M* = 262.9, *SE* = 6.50; regular in DT: *M* = 267.7, *SE* = 5.44; all *p* > 0.084).

The parietal P300 mean amplitude showed an effect of stimulus in all four 100-ms time windows spanning across the entire 300–700 ms period (all *p* < 0.001) indicating greater amplitude for the target compared to the regular stimulus (values of the four time windows for target stimuli: *M* = 1.46, *SE* = 0.25; *M* = 1.95, *SE* = 0.25; *M* = 1.60, *SE* = 0.19; *M* = 0.88, *SE* = 0.19; and for regular stimuli: *M* = 0.28, *SE* = 0.18; *M* = -0.45, *SE* = 0.16; *M* = -0.07, *SE* = 0.14; *M* = -0.02, *SE* = 0.14. The parietal P300 amplitude also showed a main effect of group (*p* = 0.034) at 600–700 ms post-stimulus window, indicating a greater amplitude for the PD group (*M* = 0.74, *SE* = 0.20) compared to the healthy group (*M* = 0.13, *SE* = 0.20) of participants.

## Discussion

This study investigated behavioral and EEG outcomes associated with cognitive-motor dual-tasking while maintaining balance in a semi-tandem stance in people in the early stages of PD and healthy controls.

### Behavioral measures

Participants with PD demonstrated reduced postural sway compared to controls, with sway further decreasing during dual-task (DT) conditions compared to single-task (ST) conditions. In other words, the less challenging health status and task condition were associated with increased/faster sway during static balancing, which could indicate that such a sway profile reflects a more flexible and efficient balance control system^53^. The reduced and slower postural sway in PD participants could stem from elevated tonic postural tone and impaired automatic postural responses to external disturbances^54^. Although dopamine therapy generally alleviates increased tonic tone, it has been shown to further compromise automatic postural responses^18^. Consequently, decreased center of pressure (COP) displacements may signify the bradykinesia and rigidity commonly seen in PD. Nevertheless, PD is a hypokinetic disorder characterized by decreased rather than increased body movements, which is also supported by our COP data. Our results are in line with previous literature showing greater postural sway in healthy participants than in those with PD^18,19^, though contrasting findings have also been reported, showing increased sway in participants with PD^8–14^. Importantly, however, the reduced postural sway might not reflect improved balance control for participants with PD.

The reduced postural sway observed in the DT condition may indicate enhanced automatic control of posture, likely due to the continuous cognitive task drawing attentional resources away from the postural task^55^. This effect may also align with the common resource pool theory, which suggests that cognitive and motor systems share limited attentional resources, with part of this capacity engaged by the cognitive task during dual-tasking^56–60^.

### Spectral power

Spectral analyses of the EEG signal revealed distinct patterns of power distribution across frequency bands and task conditions, however, no distinction was observed between healthy controls and participants with PD. Slow oscillations (δ, θ) showed more synchronization in conditions constituting a cognitive task, whether in ST or DT form, compared to the single-task balancing condition, across the entire midline (Fz, Cz, Pz, Oz). Conversely, more desynchronization in the β band was observed in conditions characterized by the motor demand (either in ST or DT form) compared to the single-task cognitive condition at several midline sites (Fz, Cz, Pz). Notably, increased α desynchronization at the parietal site (Pz) emerged as a distinctive feature of the cognitive-motor DT condition, highlighting a neural sensitivity to dual-task processing not present in the isolated STs.

The increase in θ power aligns with existing literature associating this frequency band with cognitive effort and workload demands, particularly in frontal θ activity, which is considered crucial for cognitive control processes (for a review, see^61^). These processes encompass various control strategies, such as reactive, proactive, inhibitory, and conflict resolution mechanisms^62^. The studies employing the cognitive-motor DT paradigms with various balancing tasks further demonstrate increased frontal θ involvement in managing cognitive-motor integration and task coordination^46,52^ as well as in response to motor task difficulty escalations, such as moving from a regular to a tandem stance^63^. A similar activation pattern across tasks was observed for δ power, which has also previously been implicated with increases in task difficulty within cognitive-motor DT balancing paradigms. For instance, heightened δ and θ power in a challenging DT balancing paradigm involving combined visual and auditory cognitive tasks, as opposed to DTs involving only a single cognitive modality, across both young and older adults were reported^64^. Low-frequency oscillations have thus been suggested as mechanisms that respond to heightened task demands and support long-range communication between distant brain regions, including higher-order frontal motor areas^65^. Furthermore, Ozdemir and colleagues^66^, suggested that δ activity might be more sensitive to postural as opposed to cognitive challenges, which, however, is not supported by our data; here, the engagement in the cognitive rather than the motor task drove the observed results of increased δ and θ power which suggests that cognitive compared to postural task difficulty might have a higher influence in determining the overall task difficulty during balancing.

The frequency band that did show a better sensitivity to postural challenges in this study was the β band. Decreased β power, or increased β desynchronization, is well-documented in relation to motor planning and execution, with a β rebound typically following the completion of movement (for a review, see^67^). Here, we observed a decrease in β power under conditions requiring continuous motor engagement to maintain an upright semi-tandem stance, whereas the single-task cognitive condition (without postural demands) showed increased β power. This pattern suggests that β desynchronization may facilitate essential balance control processes, including sensory reception and integration, motor command generation, and musculoskeletal responses^68^. Furthermore, cyclic β desynchronization and synchronization have been shown to align with distinct phases of the gait cycle, reinforcing β’s role in coordinating full-body movements^69^. This study provides evidence that medicated participants with PD also rely on β desynchronization to maintain balance in a complex cognitive-motor DT paradigm.

The α band also showed notable power reductions over central and posterior regions during dual-tasking. A recent study on healthy older adults reported similar α-band suppression during DT standing combined with a serial-3 subtraction task, as compared to single-task standing^70^. Other lab-based studies linked α activity (8–12 Hz) with visual-attentional demands in dynamic settings, observing α suppression over parietal, occipital, and temporal sites when participants turned rather than walked straight^71,72^, or while DT-walking compared to DT-standing^73^. These studies suggest that executive control demands, particularly those requiring inhibitory processes, may influence α activity^71,72^. Consistent with this, we observed increased α desynchronization specific to cognitive-motor DT conditions, suggesting that α desynchronization is particularly responsive to dual-task demands rather than to isolated task performance.

### Event-related components

Early visual components have been implicated in orienting visual attention. Specifically, the literature on visual spatial attention demonstrates N1 amplitude enhancement upon directing attention to the location of a stimulus, suggesting a gain control mechanism (for a review, see^74^). Similarly, enhanced N1 amplitudes were observed in the cognitive-motor DT balancing study while performing either spatial or non-spatial cognitive tasks in young adults^75^, indicating enhancement of selective attention in the upright posture. Within this context, our data suggest selective amplification of sensory processing for the DT target trials compared to both ST target trials and DT regular trials.

 The frontocentral stimulus-locked components were extracted to investigate the neurophysiological mechanisms of executive function using word-color Stroop stimuli, necessitating selective processing amidst potential distractions. Here, we observed greater P2 amplitude for processing of target stimuli compared to regular stimuli, but only in participants with PD. We also observed longer P2 latencies for processing targets as compared to regular stimuli and longer P2 latencies were also observed in participants with PD compared to healthy controls. Increasing the cognitive load in the cognitive-motor DT paradigms previously demonstrated the association with increased P2 amplitude and longer P2 latencies^46^. The P2 component has been linked to executive function, especially working memory^76–78^. Our findings support that target stimuli processing and PD-related stimuli processing are associated with greater involvement of working memory processes. The effects observed in participants with PD possibly reflect neural compensatory mechanisms^79^ or inefficient neural processing (for their interrelated roles, see^80^).

The frontocentral N2 amplitude that follows the P2 deflection is suggested to reflect mechanisms of conflict monitoring during interference control (for a review, see^81^). The present study shows greater negativity for regular stimuli compared to target stimuli. A similar effect was observed by Peskar & colleagues^46^, where the least challenging Stroop task variation elicited the greatest N2 deflection, and by Kousaie & Phillips^82^, where the larger N2 amplitude during the Flanker task arguably reflected the processing of target-irrelevant information, namely the flankers. Therefore, a reduced N2 amplitude in response to target stimuli may signify a selective focus on task-relevant information, indicating effective interference control.

The final component examined was the parietal P300 mean amplitude. In oddball paradigms, target stimuli typically elicit a more pronounced P300 response than regular stimuli^60,83^, as was observed here across the 300–700 ms post-stimulus time window. This effect is commonly associated with stimulus evaluation and decision-making processes^84,85^. Mobile brain/body imaging (MoBI;^86–88^) studies have also linked the P300 component to resource availability because it tends to diminish under conditions of increased motor load, such as walking when compared to sitting or standing^57,52,73,89,90^. In our study, however, comparing sitting to standing, we observed no such effect, which is in line with the results observed by Peskar et al.^46^; note, however, that they observed a trend for decreased P300 amplitude during standing and walking compared to sitting). This could indicate that the additional cortical resources recruited to accommodate standing during DT did not exceed the limited resources available.

For the later time windows, roughly 500 - 700 ms post-stimulus, participants with PD demonstrated greater P300 mean amplitude compared to healthy participants. Sometimes labelled the late positive complex (LPC), this component is associated with conflict resolution and response selection^91–95^. In our previous work^46^, the LPC demonstrated sensitivity to cognitive load manipulation in cognitive-motor DT and was elevated 500 - 700 ms post-stimulus during the most difficult Stroop task variant, suggesting prolonged conflict processing. Our current findings suggest that the neurodegeneration caused by PD may similarly increase demands on conflict resolution processes, comparable to increasing cognitive load. This interpretation aligns with findings obtained in Alzheimer’s Disease and more advanced PD stages, where neurodegeneration similarly impacts conflict resolution^33,34^.

## Limitations

Our participants with PD were evaluated while on medication, suggesting that antiparkinsonian drugs may have influenced the observed effects. For instance, dopamine therapy has been shown to mitigate several features associated with neurodegeneration, such as shortening the typically prolonged P300 peak^96^, and reducing elevated tonic background postural tone^54^. Furthermore, the variability in medication regimens across participants, including frequency, number of medications, and differing drug actions, may have introduced additional noise into the data, potentially impacting our findings. Future studies would benefit from examining balance control in states both on- and off-medication.

Finally, using an overt response method for the cognitive task could improve control over behavioral outcomes. Although performing the practice runs should eliminate any difficulties in understanding the task instructions, we cannot rule out the possibility that the task was not performed in accordance with the instructions across conditions and groups. Future research should consider implementing a more direct response method to enhance the accuracy and interpretability of task performance.

## Conclusion

In summary, this study indicates that postural instability in PD is characterized primarily by rigidity, manifested as reduced postural sway, rather than by excessive sway. In the EEG data, we observed increased spectral power in low-frequency bands in response to cognitive load, enhanced β desynchronization in response to motor demands, and greater α desynchronization specifically in dual-task (DT) conditions for both healthy participants and those with PD. Additionally, ERP markers associated with working memory and conflict resolution showed heightened or prolonged effort in participants with PD, potentially reflecting compensatory or inefficient neural processes.

Balance is a multifaceted capability, reliant on the coordinated integration of various sensory and motor processes. EEG, with its high temporal precision, offers an invaluable tool for monitoring these control processes at the cortical level, making it an increasingly indispensable method for studying disease-related balance impairments and fall prevention. When combined with kinematic tracking and electromyography, mobile EEG allows for a more precise analysis of disease-related deviations from healthy function^68^. This integrative approach can help pinpoint specific impairments, such as declines in sensory acuity, cognitive limitations, delays between cortical processing and movement initiation^97^, or biomechanical challenges such as reduced muscle strength. The current findings highlight reduced cognitive capacities in early PD, particularly in selective attention, rather than declines in sensory acuity, underscoring the importance of addressing cognitive factors early in intervention strategies for PD.

## Supplementary Information

Supplementary Information.

## Data Availability

The datasets used for the present study will be made available from the corresponding author upon reasonable request.
