# Supplementary material for: Neurophysiological underpinnings of balance control and cognitive-motor interaction in early Parkinson’s disease
Source: Sci Rep. 2025 Jul 11;15:25082. doi: 10.1038/s41598-025-06777-1 (PMC12254356; doi:10.1038/s41598-025-06777-1)
Supplement: Supplementary file 1 — Supplementary Information. [file 41598_2025_6777_MOESM1_ESM.docx]

Table S1 Mixed between-within ANOVA results for the spectral power of six frequency bands.

| SITE | FREQUENCY | FACTOR | *df_1,_ df_2_* | F | *p* |  | *adj*$\boldsymbol{\eta}_{\mathbf{p}}^{\mathbf{2}}$ | |
| --- | --- | --- | --- | --- | --- | --- | --- | --- |
| Fz | **δ** | Group | 1, 34 | .53 | .470 |  | -0.014 |  |
|  |  | Task | 2, 68 | 15.3 | **< .001** |  | **0.291** |  |
|  |  | G*T | 2, 68 | .02 | .977 |  | -0.028 |  |
|  | **θ** | Group | 1, 34 | 2.09 | .157 |  | 0.030 |  |
|  |  | Task | 1.4, 48 | 38.3 | **< .001** | _(GG)_ | **0.515** |  |
|  |  | G*T | 1.4, 48 | .26 | .696 | _(GG)_ | -0.021 |  |
|  | **α** | Group | 1, 34 | 1.71 | .200 |  | 0.020 |  |
|  |  | Task | 1.5, 51 | .46 | .572 | _(GG)_ | -0.016 |  |
|  |  | G*T | 1.5, 51 | 2.05 | .150 | _(GG)_ | 0.029 |  |
|  | **β** | Group | 1, 34 | .72 | .403 |  | -0.008 |  |
|  |  | Task | 2, 68 | 2.60 | **.082** |  | **0.044** |  |
|  |  | G*T | 2, 68 | .10 | .905 |  | -0.026 |  |
|  | **γ-low** | Group | 1, 34 | .01 | .947 |  | -0.029 |  |
|  |  | Task | 2, 68 | .14 | .872 |  | -0.025 |  |
|  |  | G*T | 2, 68 | .64 | .532 |  | -0.011 |  |
|  | **γ-high** | Group | 1, 34 | .12 | .737 |  | -0.026 |  |
|  |  | Task | 2, 68 | .01 | .990 |  | -0.029 |  |
|  |  | G*T | 2, 68 | .70 | .498 |  | -0.009 |  |
| Cz | **δ** | Group | 1, 34 | .41 | .525 |  | -0.017 |  |
|  |  | Task | 2, 68 | 4.81 | **.011** |  | **0.098** |  |
|  |  | G*T | 2, 68 | .19 | .829 |  | -0.023 |  |
|  | **θ** | Group | 1, 34 | 1.50 | .230 |  | 0.014 |  |
|  |  | Task | 1.5, 50.4 | 17.3 | **< .001** | _(GG)_ | **0.317** |  |
|  |  | G*T | 1.5, 50.4 | .25 | .709 | _(GG)_ | -0.023 |  |
|  | **α** | Group | 1, 34 | 1.82 | .187 |  | 0.023 |  |
|  |  | Task | 1.5, 49.8 | 1.23 | .293 | _(GG)_ | 0.006 |  |
|  |  | G*T | 1.5, 49.8 | .59 | .506 | _(GG)_ | -0.013 |  |
|  | **β** | Group | 1, 34 | .41 | .526 |  | -0.017 |  |
|  |  | Task | 2, 68 | 3.15 | **.049** |  | **0.058** |  |
|  |  | G*T | 2, 68 | .59 | .553 |  | -0.012 |  |
|  | **γ-low** | Group | 1, 34 | .03 | .876 |  | -0.028 |  |
|  |  | Task | 1.5, 49.8 | .05 | .916 | _(GG)_ | -0.029 |  |
|  |  | G*T | 1.5, 49.8 | 1.23 | .290 | _(GG)_ | 0.006 |  |
|  | **γ-high** | Group | 1, 34 | .19 | .670 |  | -0.024 |  |
|  |  | Task | 1.5, 51.2 | .368 | .634 | _(GG)_ | -0.018 |  |
|  |  | G*T | 1.5, 51.2 | 1.29 | .278 | _(GG)_ | 0.008 |  |
| Pz | **δ** | Group | 1, 34 | .31 | .439 |  | -0.011 |  |
|  |  | Task | 2, 68 | 12.1 | **< .001** |  | **0.241** |  |
|  |  | G*T | 2, 68 | .41 | .667 |  | -0.017 |  |
|  | **θ** | Group | 1, 34 | 2.15 | .152 |  | 0.031 |  |
|  |  | Task | 1.4, 49.1 | 16.7 | **< .001** | _(GG)_ | **0.311** |  |
|  |  | G*T | 1.4, 49.1 | 1.78 | .187 | _(GG)_ | 0.023 |  |
|  | **α** | Group | 1, 34 | 1.52 | .225 |  | 0.015 |  |
|  |  | Task | 1.5, 50 | 4.5 | **.025** | _(GG)_ | **0.092** |  |
|  |  | G*T | 1.5, 50 | 2.64 | **.096** | _(GG)_ | **0.044** |  |
|  | **β** | Group | 1, 34 | .47 | .497 |  | -0.015 |  |
|  |  | Task | 2,68 | 10.4 | **< .001** |  | **0.213** |  |
|  |  | G*T | 2,68 | 1.81 | .172 |  | 0.022 |  |
|  | **γ-low** | Group | 1, 34 | .52 | .475 |  | -0.014 |  |
|  |  | Task | 2,68 | .34 | .713 |  | -0.019 |  |
|  |  | G*T | 2,68 | .19 | .828 |  | -0.023 |  |
|  | **γ-high** | Group | 1, 34 | .39 | .539 |  | -0.018 |  |
|  |  | Task | 2,68 | 1.02 | .365 |  | 0.000 |  |
|  |  | G*T | 2,68 | .14 | .872 |  | -0.025 |  |
| Oz | **δ** | Group | 1, 34 | .08 | .782 |  | -0.027 |  |
|  |  | Task | 1.7, 56.9 | 10.54 | **< .001** | _(GG)_ | **0.214** |  |
|  |  | G*T | 1.7, 56.9 | .07 | .900 | _(GG)_ | -0.028 |  |
|  | **θ** | Group | 1, 34 | 1.62 | .211 |  | 0.018 |  |
|  |  | Task | 1.3, 43.2 | 25.84 | **< .001** | _(GG)_ | **0.420** |  |
|  |  | G*T | 1.3, 43.2 | .53 | .512 | _(GG)_ | -0.015 |  |
|  | **α** | Group | 1, 34 | 1.67 | .204 |  | 0.019 |  |
|  |  | Task | 1.4, 49.0 | .07 | .874 | _(GG)_ | -0.027 |  |
|  |  | G*T | 1.4, 49.0 | .36 | .632 | _(GG)_ | -0.018 |  |
|  | **β** | Group | 1, 34 | .12 | .735 |  | -0.026 |  |
|  |  | Task | 1.7, 56.4 | .01 | .994 | _(GG)_ | -0.030 |  |
|  |  | G*T | 1.7, 56.4 | .46 | .601 | _(GG)_ | -0.017 |  |
|  | **γ-low** | Group | 1, 34 | .01 | .915 |  | -0.029 |  |
|  |  | Task | 1.6, 55.8 | 2.22 | .127 | _(GG)_ | 0.034 |  |
|  |  | G*T | 1.6, 55.8 | .76 | .447 | _(GG)_ | -0.006 |  |
|  | **γ-high** | Group | 1, 34 | .01 | .958 |  | -0.029 |  |
|  |  | Task | 1.6, 54.0 | 2.81 | **.080** | _(GG)_ | **0.049** |  |
|  |  | G*T | 1.6, 54.0 | .60 | .581 | _(GG)_ | -0.012 |  |

Abbreviation: δ – delta, θ – theta, α – alpha, β – beta, γ-low – low gamma, γ-high – high gamma, G*T – group*task interaction, GG – Greenhouse Geiser corrected, *adj*$\eta_{p}^{2}$ – adjusted partial eta squared.

Table S2 Mixed between-within ANOVA results for the spectral power of six frequency bands.

| SITE | COMPONENT | FACTOR | F | *p* | *adj*$\boldsymbol{\eta}_{\boldsymbol{p}}^{\boldsymbol{2}}$ |
| --- | --- | --- | --- | --- | --- |
| Oz | N1 amplitude | Group | 2.21 | .146 | 0.033 |
|  |  | Task | 4.23 | **.047** | 0.085 |
|  |  | Stimulus | .30 | .588 | -0.020 |
|  |  | G*T | .55 | .462 | -0.013 |
|  |  | G*S | .54 | .468 | -0.013 |
|  |  | T*S | 8.37 | **.007** | **0.173** |
|  |  | G*T*S | .08 | .778 | -0.027 |
|  | N1 latency | Group | 1.88 | .180 | 0.024 |
|  |  | Task | 2.86 | .100 | 0.051 |
|  |  | Stimulus | .01 | .918 | -0.029 |
|  |  | G*T | 1.27 | .268 | 0.008 |
|  |  | G*S | 2.13 | .154 | 0.031 |
|  |  | T*S | 2.22 | .145 | 0.033 |
|  |  | G*T*S | .28 | .598 | -0.021 |
| Oz | P2 amplitude | Group | 2.34 | .135 | 0.036 |
|  |  | Task | .20 | .655 | -0.023 |
|  |  | Stimulus | 2.02 | .164 | 0.028 |
|  |  | G*T | .04 | .839 | -0.028 |
|  |  | G*S | .26 | .616 | -0.022 |
|  |  | T*S | .35 | .561 | -0.019 |
|  |  | G*T*S | .63 | .433 | -0.011 |
|  | P2 latency | Group | .54 | .467 | -0.013 |
|  |  | Task | .74 | .396 | -0.008 |
|  |  | Stimulus | 3.85 | .058 | 0.076 |
|  |  | G*T | .21 | .646 | -0.023 |
|  |  | G*S | .07 | .800 | -0.027 |
|  |  | T*S | .58 | .452 | -0.012 |
|  |  | G*T*S | .01 | .927 | -0.029 |
| FCz | P2 amplitude | Group | 1.40 | .245 | 0.011 |
|  |  | Task | .90 | .350 | -0.003 |
|  |  | Stimulus | 2.39 | .131 | 0.039 |
|  |  | G*T | .30 | .585 | -0.020 |
|  |  | G*S | 5.04 | **.031** | **0.103** |
|  |  | T*S | 2.07 | .160 | 0.029 |
|  |  | G*T*S | .01 | .910 | -0.029 |
|  | P2 latency | Group | 11.1 | **.002** | **0.224** |
|  |  | Task | 1.76 | .193 | 0.021 |
|  |  | Stimulus | 6.41 | **.016** | **0.134** |
|  |  | G*T | .60 | .444 | -0.012 |
|  |  | G*S | .85 | .364 | -0.005 |
|  |  | T*S | .33 | .572 | -0.020 |
|  |  | G*T*S | .007 | .936 | -0.029 |
| FCz | N2 amplitude | Group | 1.43 | .241 | 0.012 |
|  |  | Task | 1.49 | .231 | 0.014 |
|  |  | Stimulus | 5.44 | **.026** | **0.113** |
|  |  | G*T | .449 | .507 | -0.016 |
|  |  | G*S | .16 | .697 | -0.024 |
|  |  | T*S | .10 | .756 | -0.026 |
|  |  | G*T*S | .75 | .392 | -0.007 |
|  | N2 latency | Group | .84 | .365 | -0.005 |
|  |  | Task | .35 | .558 | -0.019 |
|  |  | Stimulus | .09 | .769 | -0.026 |
|  |  | G*T | .01 | .918 | -0.029 |
|  |  | G*S | .242 | .626 | -0.022 |
|  |  | T*S | 4.76 | **.036** | **0.097** |
|  |  | G*T*S | .75 | .392 | -0.007 |
| Pz | Mean Amplitude | Group | .67 | .418 | -0.010 |
|  | 300-400 | Task | .68 | .416 | -0.009 |
|  |  | Stimulus | 30.4 | **<.001** | **0.456** |
|  |  | G*T | .134 | .717 | -0.025 |
|  |  | G*S | .88 | .355 | -0.004 |
|  |  | T*S | .17 | .681 | -0.024 |
|  |  | G*T*S | .28 | .598 | -0.021 |
|  | Mean Amplitude | Group | 2.2 | .144 | 0.034 |
|  | 400-500 | Task | .91 | .347 | -0.003 |
|  |  | Stimulus | 87.6 | **<.001** | **0.712** |
|  |  | G*T | .05 | .828 | -0.028 |
|  |  | G*S | .24 | .627 | -0.022 |
|  |  | T*S | .00 | .986 | -0.029 |
|  |  | G*T*S | .02 | .891 | -0.028 |
|  | Mean Amplitude | Group | 3.37 | .075 | 0.063 |
|  | 500-600 | Task | .84 | .367 | -0.005 |
|  |  | Stimulus | 81.7 | **<.001** | **0.697** |
|  |  | G*T | 1.65 | .207 | 0.018 |
|  |  | G*S | .92 | .345 | -0.003 |
|  |  | T*S | .028 | .868 | -0.028 |
|  |  | G*T*S | 2.14 | .152 | 0.031 |
|  | Mean Amplitude | Group | 4.86 | **.034** | **0.099** |
|  | 600-700 | Task | .56 | .458 | -0.013 |
|  |  | Stimulus | 27.2 | **<.001** | **0.429** |
|  |  | G*T | .04 | .842 | -0.028 |
|  |  | G*S | 2.64 | .113 | 0.045 |
|  |  | T*S | .012 | .915 | -0.029 |
|  |  | G*T*S | 2.09 | .158 | 0.030 |

Note: In all cases, *df_1_ =* 1, and *df_2_* = 34. Abbreviation: G*T – group*task interaction, G*S – group*stimulus interaction, T*S – task*stimulus interaction, G*T*S – group*task*stimulus interaction, *adj*$\eta_{p}^{2}$ – adjusted partial eta squared..
